# A Comprehensive Review: The Bidirectional Role of Sebum in Skin Health

**DOI:** 10.3390/bioengineering12121333

**Published:** 2025-12-06

**Authors:** Dan Li, Ziyan Zhou, Xiaobin Yang, Qirong Zhang, Jiaming Xu, Christos C. Zouboulis, Qi Xiang, Shu Zhang

**Affiliations:** 1 Guangdong Provincial Key Laboratory of Pharmaceutical Preparations Research and Evaluation, School of Pharmacy, Guangdong Pharmaceutical University, Guangzhou 510006, China; 2112440212@stu.gdpu.edu.cn; 2 Biopharmaceutical R&D Center, Jinan University, Guangzhou 510632, China; yxbpost@163.com (X.Y.); zhangqq321@outlook.com (Q.Z.); 3 State Key Laboratory of Bioactive Molecules and Druggability Assessment, Jinan University, Guangzhou 510632, China; a13579bcd@163.com; 4 Shenzhen Yusu Biotechnology Co., Ltd., Shenzhen 518000, China; xujiaming@lege-inc.com; 5 Departments of Dermatology, Venereology, Allergology and Immunology, Brandenburg Medical School, Staedtisches Klinikum Dessau, Auenweg 38, 06847 Dessau, Germany; christos.zouboulis@mhb-fontane.de

**Keywords:** bidirectional role of sebum, sebaceous glands, molecular mechanism, dry skin, oily skin, acne vulgaris

## Abstract

Sebum plays a dual role in skin homeostasis, maintaining barrier function and providing antimicrobial defense. However, its dysregulation drives the pathophysiology of common skin diseases. This review explains that abnormal sebum secretion promotes acne vulgaris by inducing follicular occlusion and *Cutibacterium acnes* (*C. acnes*) proliferation, while altered composition impairs the epidermal barrier. Key factors such as high-glycemic diets, androgen fluctuations, and environmental conditions significantly influence sebaceous gland activity. The underlying molecular mechanisms involve inflammatory and hormonal pathways, including dysregulation of peroxisome proliferator-activated receptor gamma (PPARγ) and nuclear factor kappa-light-chain-enhancer of activated B cells (NF-κB) signaling. We conducted a systematic literature search using keywords related to sebum physiology and pathology. This review also discusses emerging therapeutic targets and the potential of advanced sebaceous gland models for future research. This synthesis provides a theoretical foundation for developing targeted interventions in dermatology and cosmetics.

## 1. Introduction

Sebum, a lipid mixture secreted by sebaceous glands, serves multiple physiological roles in skin homeostasis. It forms a hydrophobic film that reduces transepidermal water loss (TEWL), maintains stratum corneum hydration, and contains antimicrobial lipids that inhibit pathogenic microbial colonization [1]. It also helps coordinate a homeostatic perifollicular inflammatory infiltrate [2]. However, the precise regulation of sebum secretion is crucial, as both excess and deficiency can lead to distinct skin disorders. This biological duality highlights the importance of understanding sebum’s bidirectional influence on skin health.

Clinical data reveal that approximately 25% of adults exhibit an oily skin phenotype characterized by sebum secretion rates exceeding 1.5 mg/cm^2^/3 h [3], while mixed skin accounts for 50% of cases, reflecting significant regional heterogeneity. The global burden of sebum-related disorders is substantial. Acne, for instance, is the eighth most prevalent disease worldwide, with an estimated incidence of 9.4% [4,5]. Seborrheic dermatitis is another common condition, affecting approximately 3–5% of the general population [6]. Dysregulated sebum secretion represents a widespread health issue that significantly impacts patients’ quality of life and psychological well-being.

The pathological mechanisms underlying these conditions are primarily driven by sebum imbalance. Excessive sebum not only disrupts stratum corneum lipid ratios but also precipitates abnormal follicular duct keratinization and microbiota dysbiosis, thereby promoting *C. acnes* over-proliferation, inflammatory cytokine release, and the formation of comedones and inflammatory lesions. This exacerbates conditions such as acne, seborrheic dermatitis, and androgenetic alopecia [4,7]. Conversely, sebaceous hyposecretion compromises epidermal permeability barrier function, elevating susceptibility to pathologies such as atopic dermatitis, psoriasis, and hidradenitis suppurativa [8].

Therefore, this study aimed to systematically elucidate the physiological and pathological mechanisms underlying sebaceous gland function and, based on current evidence, to explore innovative treatment strategies specifically targeting oily skin and its associated disorders.

## 2. Methods

### 2.1. Literature Search Strategy

A structured and comprehensive literature search was conducted to identify relevant studies for this narrative review. The electronic databases PubMed, Web of Science, and Scopus were searched from inception until October 2025. The search strategy used a combination of keywords and MeSH terms related to the core concepts: (sebum OR sebaceous gland OR skin lipid) AND (regulat OR secret OR physiology OR homeostasis) AND (acne OR seborrheic dermatitis OR oily skin OR dysregulation). The search was designed to be broad and inclusive, focusing primarily on human studies but also considering in vitro models providing key mechanistic insights. No language restrictions were formally applied, although the final synthesis primarily reflects findings reported in the English-language literature.

### 2.2. Screening and Study Selection

The retrieved records were screened for relevance by multiple authors based on their titles and abstracts, followed by a full-text assessment of potentially eligible articles. Studies were selected for inclusion based on their contribution to understanding the physiological and pathological roles of sebum. Importantly, because this was a narrative synthesis, a formal quality assessment or a PRISMA-style systematic screening process was not employed. The overarching goal was to ensure a robust and representative overview of the current evidence base.

## 3. Physiology of Sebum and Sebaceous Glands

### 3.1. Physiology of Sebaceous Glands

Sebaceous glands, ubiquitously distributed in the reticular dermis—except on the palms and soles—are anatomically linked to hair follicles, forming pilosebaceous units (PSUs). These glands exhibit considerable regional density variation, ranging from approximately 900 glands/cm^2^ on the face to fewer than 50 glands/cm^2^ on the forearm [9]. Structurally composed of acini (featuring undifferentiated, early differentiated, advanced differentiated, fully differentiated, and mature sebocytes) and excretory ducts, sebaceous glands synthesize and release sebum via holocrine secretion over a cycle of approximately 4 weeks [10,11].

### 3.2. Characteristics of Sebum

**Figure 1:**
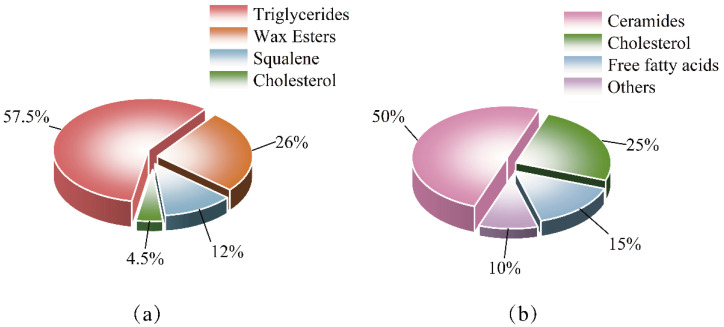
Comparative lipid composition of sebum and the epidermal stratum corneum. (**a**) The typical composition of human skin surface sebum, predominantly comprising triglycerides, wax esters, and squalene. (**b**) The characteristic lipid profile of the stratum corneum, where ceramides, free fatty acids, and cholesterol form the solid-phase barrier matrix. This fundamental difference underscores the complementary roles of sebaceous glands and keratinocytes in skin homeostasis.

Sebum and epidermal lipids possess distinct compositions that underpin their unique functions in skin homeostasis. As detailed in Figure 1a, sebum is predominantly composed of triglycerides, wax esters, and squalene, forming a fluid, hydrophobic surface film [12,13]. In contrast, the epidermal lipids of the stratum corneum are characterized by a high prevalence of ceramides, free fatty acids, and cholesterol, which create a solid-phase barrier matrix [14] (Figure 1b). This fundamental difference highlights the specialized, complementary roles of sebaceous glands and keratinocytes in maintaining skin integrity.

## 4. Factors Affecting Sebum Secretion

The regulation of sebum secretion is a complex process influenced by multiple factors, including diet, genetic factors, age-related hormonal fluctuations, environmental conditions, lifestyle, and skincare habits. These findings underscore the multifactorial nature of sebum regulation and emphasize the importance of integrating both internal and external influencing factors in clinical practice to adopt personalized strategies for modulating sebaceous gland activity.

### 4.1. Internal Factors: Molecular and Cellular Regulation of Sebum Secretion

**Figure 2:**
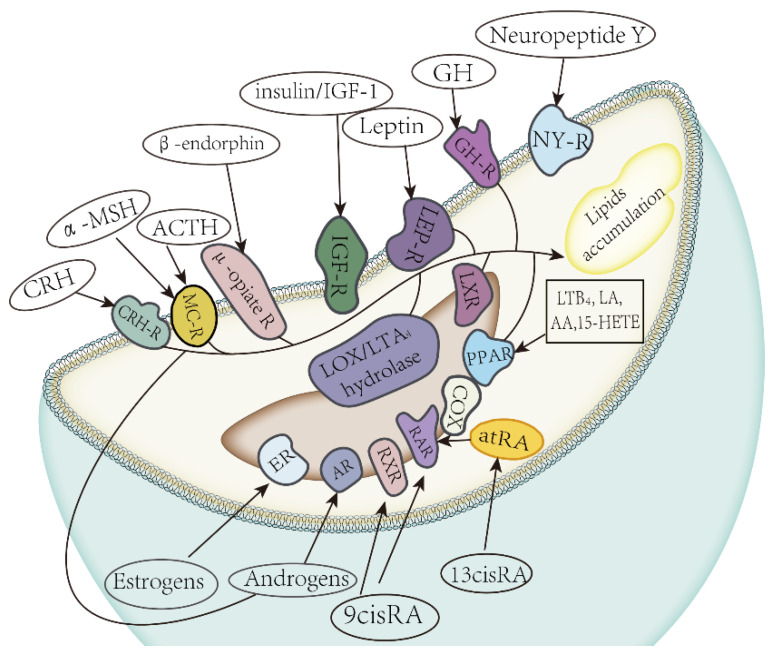
Molecular network regulating lipid accumulation in human sebocytes. The schematic illustrates the interplay of hormones, nuclear receptors, enzymes, and signaling pathways that modulate sebocyte activity and lipogenesis. R, receptor; CRH, corticotrophin-releasing hormone; α-MSH, α-melanocyte-stimulating hormone; ACTH, adrenocotricotropic hormone; 9cisRA, 9-cis retinoic acid; LTB, leukotriene; AA, arachidonic acid; LA, linoleic acid; GH, growth hormone; NY, neuropeptide Y; IGF-1, insulin-like growth factor-1; ER, estrogen receptor; AR, androgen receptor; RXR, retinoid X receptor; RAR, retinoic acid receptor; PPAR, peroxisome proliferator-activated receptor; LOX, lipoxygenase; LTA_4_, hydrolase leukotriene A4 hydrolase; COX, cyclooxygenase; LEP, Leptin; 13cisRA, 13-cis-Retinoic Acid; atRA, all-trans-Retinoic Acid; LXR, Liver X Receptor; 15-HETE, 15-Hydroxyeicosatetraenoic Acid [29].

The secretory activity of sebaceous glands is under the precise control of a sophisticated intrinsic network, encompassing endocrine signals, key transcriptional regulators, and stem cell dynamics, which collectively determine the rate and composition of sebum output. The interplay of these systems dictates the delicate balance between skin health and disease, underpinning the bidirectional nature of sebum’s role (Table 1). Figure 2 demonstrates the interplay of enzymes, membrane receptors, nuclear receptors, and their respective ligands within human sebocytes, revealing their impact on lipid accumulation.

#### 4.1.1. Hormonal Dominance: The Androgen Axis

Hormonal signals represent the most potent regulators of sebaceous activity, with the androgen axis serving as the primary mitogenic and lipogenic driver. Androgens serve as the primary mitogenic and lipogenic stimuli. They act by binding to the androgen receptor (AR) in sebocytes, activating downstream gene transcription to promote proliferation and lipid synthesis, a process markedly evident during puberty [15]. For instance, 5α-dihydrotestosterone (DHT) potently enhances triglyceride production by upregulating enzymes like acyl-CoA synthetase (ACSL) [16]. Dysregulation of androgen signaling is strongly implicated in the pathogenesis of disorders like acne and seborrheic dermatitis [17]. Other hormones, such as insulin-like growth factor-1 (IGF-1), further amplify lipid synthesis by activating the mechanistic target of rapamycin complex 1 (mTORC1) pathway, integrating nutrient signals with sebaceous activity [18].

#### 4.1.2. Nuclear Receptors: Masters of Lipogenesis and Differentiation

Beyond hormonal control, nuclear receptors function as master regulators of sebocyte differentiation and lipogenesis, with PPARγ playing a central role. The peroxisome proliferator-activated receptor (PPAR) family, particularly PPARγ, is a central driver of sebocyte terminal differentiation. Upon forming a heterodimer with retinoid X receptor (RXR), PPARγ activates a lipogenic program involving genes such as fatty acid transporter CD36 and stearoyl-CoA desaturase 1 (SCD1), facilitating the production of sebum-specific lipids like squalene and wax esters [19,20]. In contrast, retinoids, through their intracellular metabolism to all-trans retinoic acid, which binds to retinoic acid receptors (RARs), exert an opposing effect by inhibiting sebocyte proliferation and promoting ductal keratinization, thereby reducing sebum output [21].

#### 4.1.3. Stem Cells and Lineage Commitment

The physiological turnover and function of sebaceous glands rely on the dynamic equilibrium of stem cells and precise regulation by the microenvironment. Lrig1+ and Blimp1+ stem cells are located at the glandular base and maintain glandular regeneration [22]. The differentiation trajectory of these stem cells toward the sebocyte lineage is bidirectionally regulated by signaling pathways such as Wnt/β-catenin and Hedgehog (Hh) [23]. Concurrently, the Notch pathway promotes sebocyte terminal differentiation within the gland, whereas its inhibition outside the microenvironment prevents stem cell over-proliferation [24].

#### 4.1.4. Inflammation and Immune Crosstalk

The sebaceous gland is an active immunologic interface, where the composition of sebum lipids is not only crucial for barrier function but also participates in a dynamic crosstalk with the local immune system. However, compositional imbalances can trigger pathological inflammation. The Toll-like receptor (TLR)/NF-κB pathway, activated by *C. acnes* and other stimuli, mediates the release of pro-inflammatory cytokines such as IL-1β, a key driver of acne inflammation [25]. Furthermore, the sebum–microbiome interface, involving microbial metabolites like propionate and host receptors such as the aryl hydrocarbon receptor (AhR), plays an increasingly recognized role in local immune regulation [26].

#### 4.1.5. Emerging Regulators and Metabolic Adaptation

Other molecular players contribute to the fine-tuning of sebaceous function. The melanocortin system, particularly melanocortin receptor 5 (MC5R), is implicated in regulating sebum production, with its antagonism shown to inhibit gland activity [27]. As sebocytes differentiate and move toward the gland center, they experience progressive hypoxia, which activates hypoxia-inducible factor 1 alpha (HIF-1α). This triggers a metabolic reprogramming toward glycolysis and lipid storage, adapting to the low-oxygen environment and facilitating the final stages of lipid accumulation [28]. The synergy and antagonism of these diverse molecular mechanisms collectively maintain sebaceous gland homeostasis, and their dysregulation underpins a spectrum of pathological states.

In summary, the homeostatic function of the sebaceous gland relies on the synergy and antagonism of the diverse molecular mechanisms described above and shown in Table 1. The appropriate activation of pathways such as the androgen axis and PPARγ signaling drives the normal sebum production necessary for maintaining skin barrier integrity and antimicrobial defense. Conversely, the hyperactivation of these pathways—by hormonal fluctuations, nutrient signaling, or persistent inflammatory triggers (e.g., TLR/NF-κB activation)—leads to sebaceous hyperplasia, hyperseborrhea, and altered lipid composition, creating a permissive environment for inflammatory diseases like acne. On the other hand, suppression of these differentiations and lipogenic signals, as observed with retinoid therapy, can lead to sebum deficiency, resulting in skin dryness and impaired barrier function. This intricate regulatory network fundamentally explains the bidirectional role of sebum in skin physiology and pathology.

### 4.2. External Factors: Environmental, Skincare and Lifestyle Influences

In addition to intrinsic regulation, sebum secretion is significantly influenced by extrinsic factors—environmental, behavioral, dietary, and pharmacological (summarized in Table 2).

Among environmental factors, ambient temperature primarily functions through thermoregulatory mechanisms; an increase in skin temperature directly enhances the metabolic activity of sebaceous glands and reduces sebum viscosity, thereby promoting its excretion [30]. Exposure to PM 2.5 induces oxidative stress, leading to peroxidation of epidermal polyunsaturated fatty acids (e.g., linoleic acid) and generation of pro-inflammatory lipid mediators (e.g., leukotrienes, prostaglandins), which disrupt sebaceous lipid homeostasis and drive aberrant sebum production [31]. Similarly, prolonged mask-wearing fosters an occlusive, humid microenvironment that enhances sebum excretion and may perturb the cutaneous microbiome [32].

Skincare practices also modulate sebum output: barrier disruption from over-cleansing elicits compensatory sebum overproduction [33], whereas topical agents such as niacinamide and retinol suppress sebogenesis by targeting lipid synthesis and sebocyte differentiation pathways [34].

Dietary factors exert complex effects. High-calorie diets supply metabolic substrates for sebum synthesis and may elevate androgen levels [35]. Although high-glycemic diets are associated with acne via insulin/IGF-1–mediated lipogenesis [36], their direct impact on sebum secretion remains inconsistent—potentially attributable to genetic variation (e.g., GOLGA7B [37]), ethnicity, and dietary patterns [38]. Conversely, caloric restriction suppresses sebaceous lipogenesis through systemic metabolic adaptation [39].

Pharmacologically, isotretinoin potently inhibits sebocyte differentiation and lipid production via nuclear receptor activation [40]. Discontinuation of estrogen therapy may induce rebound seborrhea due to unopposed androgen signaling [41]. Emerging strategies, including TGF-β–modulating nanoemulsions, show promise in reducing sebum output [42].

## 5. Sebum-Related Skin Problems

Abnormal skin lipid metabolism is closely associated with the onset and progression of various common skin diseases. Dysregulation of lipid composition, either in quality or quantity, can disrupt epidermal barrier homeostasis, activate inflammatory signaling pathways, and mediate dysbiosis of microbial communities, thereby promoting disease progression.

Skin diseases linked to abnormal lipid secretion can be categorized into two groups according to lipid secretion status: (1) diseases caused by excessive lipid secretion, such as acne and seborrheic dermatitis (Table 3a); (2) diseases caused by insufficient lipid secretion, such as rosacea, psoriasis, atopic dermatitis, and ichthyosis (Table 3b) [8]. The pathogenesis of these conditions can be traced to downstream events triggered by either an overabundance or a deficiency of sebum, manifesting as compromised barrier integrity, dysbiosis of the cutaneous microbiome, and aberrant immune activation.

Furthermore, abnormalities in sebum secretion are implicated in several other cutaneous conditions. For instance, dyspigmentation has been associated with Malassezia colonization, compromised skin barrier integrity, and neurogenic inflammation [43]. Oily sensitive skin presents a seemingly paradoxical phenotype, potentially underpinned by mechanisms such as sebaceous gland atrophy, reduced squalene levels, and consequent enhanced oxidative stress [44,45]. In the context of skin aging, substances like porphyrins from *C. acnes* and squalene peroxide contribute to oxidative damage. This process, synergizing with inflammatory responses, promotes hyperpigmentation and photoaging [46,47].

**Table 3:** (**a**) Skin Disorders Associated with Sebaceous Hypersecretion. (**b**) Skin Disorders Associated with Sebaceous Hyposecretion.

	(a)	
Disease	Primary Pathogenesis Mechanisms	Reference
Acne	Sebum overproduction; follicular plugging; *C. acnes* proliferation; inflammation	[48,49]
Seborrheic dermatitis	Altered sebum composition (e.g., ↑ squalene); Malassezia overgrowth; barrier disruption; neurogenic inflammation	[50,51]
	(**b**)	
Rosacea	Ceramide deficiency; altered lipid ratios; barrier impairment; xerosis.	[52]
Psoriasis	Structural lipid loss; barrier failure; secondary inflammation.	[53]
Atopic dermatitis	Sebum decline; barrier leak; dysbiosis; neurosensory flare.	[54]
Ichthyosis	Genetic defect in lipid synthesis; severe sebum deficiency; scaling and barrier collapse.	[55]

↑, increased secretion or activation.

## 6. Therapeutic and Skincare Product Development

### 6.1. Establishment and Research Application of In Vitro Sebaceous Gland Models

Early investigations focused on short-term cultures of human sebaceous glands, laying the foundation for subsequent research. Building upon this, researchers successfully established primary human sebocyte cultures, enabling exploration of their functional properties [56]. A pivotal advancement in this field was the development of immortalized sebocyte cell lines that retain native sebaceous gland characteristics, providing reliable tools for in vitro studies [57]. The establishment of reliable in vitro sebaceous gland models has significantly advanced research into sebaceous gland biology and related disorders. These two-dimensional models have provided essential tools for studying sebaceous gland physiology, lipid metabolism, and drug screening.

The recent research focus has shifted toward more complex three-dimensional (3D) models and tissue engineering techniques [58,59,60]. 3D sebaceous spheroids better mimic the structure and function of native glands [61,62]. Additionally, stem-cell-based studies, as demonstrated by Wang et al., have revealed the regenerative potential of sebaceous glands [63], while the bioartificial gland developed by Chen et al. enables high-fidelity investigation of secretory functions and interactions with microorganisms (Table 4) [64].

These advanced models have deepened our understanding of the molecular mechanisms governing sebaceous gland differentiation, metabolism, and immune responses. They allow for simulating disease processes such as acne and accelerate novel therapy development. Future research efforts will focus on integrating multi-omics approaches, patient-derived cells, and microfluidic systems to construct more physiologically relevant models suitable for personalized medicine.

### 6.2. Treatment of Sebum-Related Skin Problems

The treatment of sebum-related skin problems, such as acne, seborrheic dermatitis, and rosacea, requires a multi-pronged strategy that targets excessive oil production, inflammation, and bacterial overgrowth. First-line pharmacological approaches include anti-androgens (e.g., spironolactone) to hormonally regulate gland activity [65], retinoids (e.g., topical tretinoin and oral isotretinoin) to normalize skin cell turnover and drastically reduce sebum production, and antimicrobial agents like benzoyl peroxide to target *C. acnes* [66].

Emerging therapies are focusing on novel pathways to offer more targeted solutions. These include PPARγ modulators (e.g., NAC-GED0507) designed to normalize sebum output and reduce inflammation simultaneously, and melanocortin receptor antagonists that have shown promise in preclinical models for shrinking sebaceous glands [67]. Additionally, postbiotic treatments containing microbial metabolites are being explored to calm inflammation and reinforce the skin’s natural barrier without disrupting the microbiome (Table 5) [68,69].

A successful long-term management plan must extend beyond medication. Holistic skincare is critical; harsh, stripping products can damage the skin barrier and trigger a rebound overproduction of oil [70]. Therefore, an effective regimen combines these medical treatments with personalized skincare advice centered on gentle cleansing, non-comedogenic moisturization, and sun protection. This integrated approach addresses the root causes while maintaining a healthy skin barrier to prevent recurrence.

### 6.3. Development of Novel Skincare Products and Technologies

The development of next-generation skincare products for oily and acne-prone skin is increasingly driven by a deeper understanding of sebaceous gland biology and the integration of advanced bioactive ingredients with robust efficacy data [71]. Modern formulations are moving beyond traditional oil-absorbing agents to target the molecular pathways that regulate sebum production, inflammation, and microbial dysbiosis [72].

Innovative ingredients with mechanistic evidence are now central to product development. For instance, niacinamide at concentrations of 2–5% has been shown to downregulate key lipogenic enzymes such as SCD1 and ACSL, leading to a 25–35% reduction in sebum excretion rates in clinical studies [73]. Similarly, bio-fermented postbiotics derived from Lactobacillus and Bifidobacterium strains modulate the skin microbiome and significantly reduce pro-inflammatory cytokines (e.g., IL-1β, TNF-α), enhancing barrier function and reducing acne lesions by ≥30% in recent trials [68,74]. Another promising agent, tripeptide-3, delivered via nanoemulsions, inhibits sebocyte differentiation and lipogenesis through the TGF-β pathway, with a ≥25% reduction in facial sebum observed after 4 weeks of use [42,75].

There is growing interest in re-evaluating established ingredients with broad mechanisms of action. Recent exploratory research, such as the use of a multi-enzyme complex presented in a conference poster (IFSCC Congress 2025, Poster 678) to improve closed comedones by exfoliating keratin and repairing the skin barrier [76], supports this approach. Colloidal sulfur, a broad-spectrum antimicrobial, effectively targets *C. acnes* and other skin mites (e.g., *Demodex* spp.). It addresses key acne factors by reducing hyperkeratinization, suppressing microbes, reducing inflammation [77], and regulating sebum production. Both novel enzyme formulations and traditional agents like colloidal sulfur offer promising, multifunctional treatments for sebum-related issues.

Advanced delivery systems are critical for maximizing efficacy while minimizing irritation. Nanoemulsions and microemulsions enhance the penetration of peptides, retinoids, and sulfur into sebocytes, enabling sustained release and improved comedolytic effects [42]. For instance, the ionic liquid nanoemulsion platform for tripeptide-3 delivery was initially investigated in melanoma therapy [75], but its sebum-modulating efficacy has been specifically demonstrated in dermatological studies [42,75]. Moreover, liposome-encapsulated retinol and salicylic acid have demonstrated 40% greater follicular delivery compared with conventional formulations in in vivo models [73].

Emerging clinical and preclinical evidence supports the shift toward multifunctional and microbiome-friendly formulations. The use of 3D sebaceous spheroid models has enabled high-fidelity screening of actives such as niacinamide and postbiotics, revealing the dose-dependent suppression of lipid accumulation and inflammatory markers [58,61]. In parallel, multi-omics approaches (lipidomics, metagenomics) are uncovering how specific sebum components interact with the cutaneous microbiome, informing the design of personalized skincare regimens [74,78].

Looking forward, the future of skincare development lies in precision dermatology—leveraging individual genetic, hormonal, and microbiome profiles to create highly tailored products. The integration of AI-driven skin analysis, microfluidic “skin-on-a-chip” systems, and patient-derived organoids will further accelerate the discovery of targeted ingredients and delivery platforms. By combining robust biological insights with advanced formulation technologies, the next generation of skincare products will not only manage sebum more effectively but also promote long-term skin health and barrier resilience.

## 7. Discussion

The research and development landscape for sebum-related skin conditions is undergoing a fundamental shift. The paradigm is moving from superficial oil control and generalized therapies toward a holistic strategy that targets the biological roots of sebum dysregulation, inflammation, and microbiome imbalance, while actively supporting the skin barrier. This review has highlighted the efficacy of this multi-targeted approach, from first-line pharmacological interventions to skincare formulations powered by bioactive ingredients with clearly elucidated mechanisms.

The future trajectory of dermatological skincare points toward precision medicine paradigms, shifting from generalized solutions to highly individualized approaches based on genetic predispositions, unique microbiome compositions, and specific environmental exposures [79]. To achieve this vision, several key future directions emerge. First, the application of multi-omics analyses (lipidomics, metagenomics, proteomics) will be crucial to decrypt the precise chemical dialogue between sebum composition and the cutaneous microbiome, particularly in conditions beyond acne such as aging or sensitive skin [80]. Second, advanced 3D models (e.g., sebaceous gland organoids, microfluidic “skin-on-a-chip” systems) and AI-driven image analysis will provide unprecedented insights into gland biology and enable high-fidelity screening of novel therapeutics, from PPARγ modulators to revitalized ingredients like colloidal sulfur, whose exact molecular mechanisms remain underexplored. Finally, resolving current controversies, such as the exact influence of specific dietary components on sebum composition, will require more robust, longitudinal studies. The strategic re-evaluation of established, multifunctional actives, as exemplified by both novel enzyme complexes and colloidal sulfur, alongside the development of targeted new molecules, embodies this forward-looking approach.

We are optimistic that by embracing these technologies and tackling these unresolved questions, the next decade will yield groundbreaking strategies to optimize skin health through a deeper, more holistic understanding of sebum’s bidirectional role.

## 8. Conclusions

This review has highlighted the essential role of sebum in skin barrier function, microbial defense, and inflammation. Sebum composition and secretion are regulated by hormonal, genetic, environmental, and dietary factors. Dysregulation contributes to skin disorders such as acne, seborrheic dermatitis, and atopic dermatitis.

Advances in research models, including immortalized sebocyte lines and 3D cultures, have improved the study of gland biology and therapeutic screening. Current treatments target sebum production through anti-androgens, retinoids, and PPAR modulators, while skincare products increasingly incorporate microbiome-friendly and barrier-supporting actives.

Future work should focus on the sebum–microbiome–immune interface, personalized approaches based on skin biomarkers, and novel therapeutics targeting specific pathways. To this end, future research must leverage multi-omics technologies and advanced bioengineered models to fill critical knowledge gaps, including the precise role of diet, the dynamics of the sebum–microbiome–immune axis in various skin conditions, and the optimization of both novel and repurposed active ingredients. Improved drug delivery systems and further use of engineered skin models will accelerate the development of precise treatments for sebum-related conditions.

## Figures and Tables

**Table 1:** The Bidirectional Impact of Sebum.

Sebum Output	Key Promoting Factors/Pathways	Sebum Composition	Consequences for Skin Health
Hypersecretion	↑ Androgens (AR signaling)	Altered lipid ratios: ↑ Squalene, ↓ linoleic acid	Follicular occlusion
	↑ IGF-1/mTORC1		*C. acnes* overgrowth
	↑ PPARγ activity		Pro-inflammatory milieu
	TLR/NF-κB inflammation		Oxidative stress
	High-glycemic diet		
Normal secretion	Normal hormonal levels	Optimal lipid composition	Maintains skin barrier
	Homeostatic PPARγ, Wnt, etc.		Antimicrobial activity
	Intact skin barrier		Hydration and skin integrity
	Stable microbiome		
Hyposecretion	Retinoid use (RAR signaling)	Reduced lipid diversity: Deficiency in all lipid classes	Compromised barrier (↑ TEWL)
	↓ Androgens/aging		Dysbiosis
	Caloric restriction		Susceptibility to irritation and inflammation
	Genetic disorders (e.g., Ichthyosis)		

↑, increased secretion or activation; ↓, decreased secretion or inhibition. AR, Androgen receptor; IGF-1, Insulin-like growth factor-1; mTORC1, Mechanistic target of rapamycin complex 1; PPARγ, Peroxisome proliferator-activated receptor gamma; TLR, Toll-like receptor; NF-κB, Nuclear factor kappa-light-chain enhancer of activated B-cells; RAR, Retinoic acid receptor; TEWL, Transepidermal water loss.

**Table 2:** External Factors Affecting Sebum Secretion.

Section	Category	Main Content	Reference
Environment	Temperature effects	↑ Secretion rate with temperature	[30]
	Pollutant impact	Oxidative stress and inflammation	[31]
	Mask-induced effects	Occlusion, ↑ excretion and dysbiosis	[32]
Skincare products	Barrier disruption	Compensatory sebum overproduction	[33]
	Active ingredients	Modulate differentiation/lipid pathways	[34]
Diet	Substrate sources	Provides metabolic substrates	[35,36]
	Caloric restriction	↓ Sebum production	[37,38,39]
Pharmacotherapy	Treatment with isotretinoin	Suppresses sebum production	[40]
	Hormonal misuse	Rebound seborrhea	[41]
	Nanoemulsions	↓ Sebum production through the TGF-β pathway	[42]

↑, increased secretion or activation; ↓, decreased secretion or inhibition; TGF-β, Transforming Growth Factor β.

**Table 4:** Model Research and Cell Culture of Sebaceous Glands.

Section	Category	Main Content	Reference
Early research	Short-term cultures	Foundation for studying human sebaceous gland biology	[56,57]
	SZ95 cell line	Immortalized human sebocytes retaining normal function; key tool for lipid and disease research	
3D models	3D sebaceous spheroids	Mimic native gland morphology; used for studying lipid production and disease mechanisms	[58,61]
	3D-printed scaffolds	Bioengineered constructs combining stem cells and SZ95 cells to replicate skin structure	[59]
	3D-SeboSkin model	Ex vivo skin explants for realistic sebaceous gland studies	[60]
	Epidermal stem cell grafts	Regenerate hair follicles and glands in wounds	[63]

**Table 5:** Key Pathways in Sebocyte Regulation.

Pathway	Key Components	Functions	Therapeutic Targets
AR Signaling	DHT, SRD5A1/2, AR, AREs	Promotes proliferation and lipogenesis	Anti-androgens
PPAR Signaling	PPARγ/RXR, PPRE, CD36, PLIN2	Drives differentiation and lipid storage	PPARγ modulators
mTORC1	IGF-1, PI3K, Akt, mTORC1, S6K	Integrates nutrients for anabolism	mTOR inhibitors
Wnt/β-Catenin	β-catenin, TCF/LEF	Regulates stem cell fate	Targeted therapies for tumors
TLR/NF-κB	TLR2/4, MyD88, NF-κB, IL-1β	Mediates inflammation in acne	Anti-inflammatories
Sebum-microbiome	Propionate, HDAC, AhR, IL-33	Links sebum to immune regulation	Topical metabolites

AR, androgen receptor; DHT, dihydrotestosterone; SRD5A1/2, steroid 5-alpha-reductase type 1/2; AREs, androgen response elements; PPARγ, peroxisome proliferator-activated receptor gamma; RXR, retinoid X receptor; PPRE, PPAR response element; CD36, cluster of differentiation 36; PLIN2, perilipin-2; mTORC1, mechanistic target of rapamycin complex 1; IGF-1, insulin-like growth factor 1; PI3K, phosphoinositide 3-kinase; Akt, Ak strain transforming; S6K, ribosomal protein S6 kinase; TCF/LEF, T-cell factor/lymphoid enhancer factor; TLR2/4, Toll-like receptor 2/4; MyD88, myeloid differentiation primary response 88; NF-κB, nuclear factor kappa-light-chain-enhancer of activated B cells; IL-1β, interleukin-1 beta; HDAC, histone deacetylase; AhR, aryl hydrocarbon receptor; IL-33, interleukin-33.

## Data Availability

No new data were created or analyzed in this study.

## Abbreviations

The following abbreviations are used in this manuscript:

*C. acnes*	*Cutibacterium acnes*
TEWL	Transepidermal water loss
PSUs	Pilosebaceous units
AR	Androgen receptor
DHT	Dihydrotestosterone
ACSL	Acyl-CoA synthetase
IGF-1	Insulin-like growth factor-1
PPAR	Peroxisome proliferator-activated receptor
RXR	Retinoid X receptor
PPARγ	Peroxisome proliferator-activated receptor gamma
SCD1	Stearoyl-CoA desaturase 1
RARs	Retinoic acid receptors
Hh	Hedgehog
TLR	Toll-like receptor
AhR	Aryl hydrocarbon receptor
MC5R	Melanocortin receptor 5
HIF-1α	Hypoxia-inducible factor 1 alpha
CRH	Corticotrophin-releasing hormone
α-MSH	α-Melanocyte-stimulating hormone
ACTH	Adrenocotricotropic hormone
9cisRA	9-cis retinoic acid, LTB leukotriene
AA	Arachidonic acid
LA	Linoleic acid
GH	Growth hormone
NY	Neuropeptide Y
PG	Prostaglandin
ER	Estrogen receptor
LOX	Lipoxygenase
LTA_4_	Hydrolase leukotriene A4 hydrolase
13cisRA	13-cis-Retinoic Acid
atRA	all-trans-Retinoic Acid
LXR	Liver X Receptor
15-HETE	15-Hydroxyeicosatetraenoic Acid
COX	Cyclooxygenase
ROS	Reactive oxygen species
LEP	Leptin
3D-SeboSkin	3D sebaceous skin model
SRD5A1/2	Steroid 5-alpha-reductase type 1/2
AREs	Androgen response elements
PPRE	PPAR response element
CD36	Cluster of differentiation 36
PLIN2	Perilipin-2
mTORC1	Mechanistic target of rapamycin complex 1
PI3K	Phosphoinositide 3-kinase
Akt	Ak strain transforming
S6K	Ribosomal protein S6 kinase
TCF/LEF	T-cell factor/lymphoid enhancer factor
MyD88	Myeloid differentiation primary response 88
NF-κB	Nuclear factor kappa-light-chain-enhancer of activated B cells
IL-1β	Interleukin-1 beta
HDAC	Histone deacetylase
IL-33	Interleukin-33
